# Invasive Fusariosis in Nonneutropenic Patients, Spain, 2000–2015

**DOI:** 10.3201/eid2701.190782

**Published:** 2021-01

**Authors:** Elena Pérez-Nadales, Ana Alastruey-Izquierdo, María José Linares-Sicilia, Juan Carlos Soto-Debrán, Edson Abdala, Julio García-Rodríguez, Miguel Montejo, Patricia Muñoz, Miguel Salavert Lletí, Antonio Rezusta, Maite Ruiz Pérez de Pipaón, Lucrecia Yáñez, Esperanza Merino, María Isolina Campos-Herrero, José María Costa-Mateo, Jesús Fortún, Tomás García-Lozano, Carolina Garcia-Vidal, Mario Fernández-Ruiz, Ferrán Sánchez-Reus, Carmen Castro-Méndez, Inmaculada Guerrero-Lozano, Pere Soler-Palacín, José María Aguado, Luis Martínez-Martínez, Julian Torre-Cisneros, Marcio Nucci, the Spanish Fusariosis Study Group

**Affiliations:** Spanish Network for Research in Infectious Diseases, Instituto de Salud Carlos III, Madrid, Spain (E. Pérez-Nadales, A. Alastruey-Izquierdo, M. Montejo, M. Ruiz Pérez de Pipaón, L. Yáñez, J. Fortún, C. Garcia-Vidal, M. Fernández-Ruiz, I. Guerrero-Lozano, P. Soler-Palacín, J.M. Aguado, L. Martínez-Martínez, J. Torre-Cisneros);; Maimonides Biomedical Research Institute of Cordoba, Reina Sofia University Hospital, University of Cordoba, Cordoba, Spain (E. Perez-Nadales, M.J. Linares Sicilia, J. M. Costa-Mateo, L. Martínez-Martínez, J. Torre-Cisneros);; Centro Nacional de Microbiología, Instituto de Salud Carlos III, Madrid, Spain (A. Alastruey-Izquierdo, J.C. Soto-Debrán);; Hospital das Clinicas, Universidade de São Paulo, São Paulo, Brazil (E. Abdala);; Hospital Universitario La Paz, Madrid (J. García-Rodríguez);; Hospital Universitario Cruces, Baracaldo, Spain (M. Montejo);; Hospital General Universitario Gregorio Marañón, Instituto de Investigación Sanitaria Hospital Gregorio Marañón, Universidad Complutense de Madrid, Madrid, Spain (P. Muñoz);; Hospital Universitario "12 de Octubre," Instituto de Investigación Hospital "12 de Octubre,” Universidad Complutense, Madrid, Spain (M. Fernández-Ruiz, J.M. Aguado);; Hospital Universitario y Politécnico La Fe, Valencia, Spain (M. Salavert Lletí);; Hospital Universitario Miguel Servet, Universidad de Zaragoza, Instituto de Investigación Sanitaria Aragón, Zaragoza, Spain (A. Rezusta);; Hospital Universitario Virgen del Rocío, Seville, Spain (M. Ruiz Pérez de Pipaón);; Hospital Universitario Marqués de Valdecilla, Instituto de Investigación Sanitaria Valdecilla, Santander, Spain (L. Yáñez);; Hospital General Universitario de Alicante, Spain (E. Merino);; Hospital Universitario de Gran Canaria Doctor Negrín, Las Palmas de Gran Canaria, Spain (M. I. Campos-Herrero);; Hospital Ramón y Cajal, Madrid (J. Fortún);; Fundación Instituto Valenciano de Oncología, Valencia, Spain (T. García-Lozano);; Hospital Universitario de Bellvitge, Barcelona, Spain (C. Garcia-Vidal);; Hospital de la Santa Creu i Sant Pau, Barcelona (F. Sánchez-Reus);; Hospital Universitario Virgen de Valme, Seville (C. Castro-Méndez);; Hospital Universitario Puerta del Mar, Cádiz, Spain (I. Guerrero-Lozano);; Hospital Universitari Vall d’Hebron, Barcelona (P. Soler-Palacín);; Universidade Federal do Rio de Janeiro, Rio de Janeiro, Brazil (M. Nucci)

**Keywords:** *Fusarium*, invasive fusariosis, incidence, mortality, neutropenia, fusariosis, Spain, fungi, fungal infections, mycotic diseases

## Abstract

Invasive fusariosis (IF) is associated with severe neutropenia in patients with concurrent hematologic conditions. We conducted a retrospective observational study to characterize the epidemiology of IF in 18 Spanish hospitals during 2000–2015. In that time, the frequency of IF in nonneutropenic patients increased from 0.08 cases per 100,000 admissions in 2000–2009 to 0.22 cases per 100,000 admissions in 2010–2015. Nonneutropenic IF patients often had nonhematologic conditions, such as chronic cardiac or lung disease, rheumatoid arthritis, history of solid organ transplantation, or localized fusariosis. The 90-day death rate among nonneutropenic patients (28.6%) and patients with resolved neutropenia (38.1%) was similar. However, the death rate among patients with persistent neutropenia (91.3%) was significantly higher. We used a multivariate Cox regression analysis to characterize risk factors for death: persistent neutropenia was the only risk factor for death, regardless of antifungal therapy.

## Abstract 2

Invasive fusariosis has a global increasing incidence and has emerged as a severe infection in nonneutropenic patients.

Invasive fusariosis (IF) is a fungal disease that mostly affects patients with hematologic malignancies or who have received hematopoietic cell transplants. These patients often have prolonged and profound neutropenia, low levels of T cells, or both (*1*,*2*). Despite advances in early diagnosis and treatment, IF remains associated with high morbidity and death rates (*3*,*4*).

Most studies on this fungal disease have occurred in North and South America (*4*–*9*). However, the epidemiology of IF in Europe has not been fully characterized; within Europe, most multicenter studies on IF have occurred in Italy and France (*8*,*10*–*12*). A multicenter study by the European Confederation of Medical Mycology reported 76 cases of IF during 2007–2012 (*13*). Most (60%) of these IF cases occurred in Italy but none in Spain. Previous single-center studies in Europe have reported regional differences in the distribution of *Fusarium* species and their susceptibilities to antimicrobial drugs, highlighting the importance of monitoring local epidemiologic data (*13*–*15*). We characterized the epidemiology of IF in Spain using a retrospective observational study that examined the effects of clinical and etiologic characteristics on outcomes in a cohort of 58 IF patients at hospitals in Spain.

## Methods

We conducted this study in 18 hospitals in Spain, 8 of which belong to the Spanish Network for Research in Infectious Diseases, Instituto de Salud Carlos III, in Madrid, Spain. The study was approved by Reina Sofía University Hospital Institutional Review Board (Córdoba, Spain), which waived the need to obtain written informed consent. During January 2000–December 2015, hospital staff reviewed microbiological and pathologic registries to identify cases of IF. Data were recorded in a password-protected, electronic clinical research file. We monitored the collected data for missing information, inconsistencies, and ambiguities; when necessary, we sent queries to the appropriate hospitals for clarification.

We conducted a blind review of reported cases of IF. In this study, we included only proven cases of IF according to the consensus definitions of invasive fungal diseases established by the European Organization for Research and Treatment of Cancer and the Mycoses Study Group Education and Research Consortium (*16*,*17*). IF can be proven in 4 ways: microscopic examination of a specimen obtained by needle aspiration or biopsy that documents hyphae and isolates *Fusarium* spp. in the same tissue; blood culture yielding *Fusarium* spp. alongside signs consistent with an infectious disease process; isolation of *Fusarium* spp. in a normally sterile site (excluding bronchoalveolar lavage fluid, a paranasal or mastoid sinus cavity specimen, and urine) with accompanying signs of infection; or amplification and sequencing of *Fusarium* DNA in formalin-fixed paraffin-embedded tissue (*16*,*17*).

The participating hospitals identified *Fusarium* isolates using genetic sequencing, matrix-assisted laser desorption/ionization time-of-flight (MALDI-TOF) mass spectrometry, or morphologic characteristics. In addition, all biological samples available at the time of this retrospective study (i.e., *Fusarium* isolates and specimens for biopsies) were sent to the Mycology Reference Laboratory at the Instituto de Salud Carlos III for confirmatory genetic sequencing. The isolates were freeze-dried on potato agar slants on arrival and stored in distilled water. Molecular identification was based on the translation elongation factor 1α gene (*18*). Isolates were cultured in GYEP medium (0.3% yeast extract, 1.0% peptone; Francisco Soria Melguizo S.A., http://www.f-soria.es) with 2.0% glucose (Sigma-Aldrich Inc., https://www.sigmaaldrich.com) at 30°C for 24–48 h. Genomic DNA was isolated by using an extraction procedure described previously (*19*). Internal transcribed spacer region and a portion of the translation elongation factor 1α gene region were amplified as previously described (*20*). Alignment results were obtained and edited using Lasergene MegAlign Pro software from DNASTAR, Inc. (https://www.dnastar.com). All sequences were compared with reference sequences from GenBank and MycoBank (https://www.mycobank.org) databases by using InfoQuest FP version 4.50 software (Bio-Rad Laboratories, https://www.bio-rad.com). We also used an in-house database of the Mycology Reference Laboratory. When only biopsy specimens were available, we used real-time PCR specific for *Fusarium* species as previously described (*21*). When identified cases did not have available isolates or biologic material for PCR, we reported the specific *Fusarium* species only if obtained by MALDI-TOF mass spectrometry in conjunction with histopathology. We reported the remaining IF cases in patients with compatible clinical signs and symptoms as caused by *Fusarium* spp.

We defined survival as the time between the date of diagnosis and death or end of follow-up care (i.e., 90 days). We defined the date of diagnosis as the day of the first *Fusarium*-positive culture. We defined disseminated fusariosis as the involvement of >1 noncontiguous organ. We did not consider fungemia cases as disseminated disease unless >1 organ was involved (e.g., skin, lung, or sinuses) (*3*). We defined neutropenia as a blood neutrophil count <500 cells/mm^3^ temporally related to the onset of fungal disease. We defined persistent neutropenia as cases with continued low neutrophil counts at the end of treatment or death. We defined resolution of neutropenia as a persistent recovery of blood neutrophil count >500/mm^3^ as determined by available hospital records.

We calculated the incidence of IF using total admissions in the participating hospitals as the denominator; we expressed the incidence as the number of cases per 100,000 admissions. For statistical purposes, we arbitrarily defined 2 time periods: 2000–2009 and 2010–2015. We compared incidences between the different periods by χ^2^ test using WinPepi version 11.65 (Brixton Health, http://www.brixtonhealth.com). We considered p<0.05 to be significant.

We collected variables describing patient demographic data, concurrent conditions, neutropenia, receipt of corticosteroids, clinical manifestations, diagnostic procedures, treatment, and outcome (i.e., 90-day survival). We compared categorical variables using χ^2^ or 2-tailed Fisher exact test, and we compared continuous variables using the Mann-Whitney U test. We conducted all statistical tests with SPSS Statistics 16.0 (IBM Inc., https://www.ibm.com) and R version 3.5.0 (R Foundation for Statistical Computing, https://www.r-project.org); we considered 2-sided p values <0.05 to be significant. We constructed unadjusted Kaplan–Meier curves and compared them using the log-rank test. In addition, we evaluated factors associated with 90-day survival. We evaluated the following variables by univariate and multivariable Cox regression analyses: age, sex, age-adjusted Charlson comorbidity index (*22*), underlying disease, receipt of antifungals and corticosteroids in the previous 30 days, neutropenia at diagnosis of fusariosis and at the end of follow-up, clinical manifestations, and primary treatment (monotherapy with a lipid formulation of amphotericin B, monotherapy with voriconazole, or combination treatment). We entered variables with p<0.1 by univariate analysis into the multivariate analysis; we included variables with p<0.05 by the multivariate analysis in the final model. We evaluated the prediction accuracy of the final Cox model using the area under the receiver operating characteristic curve.

## Results

### Incidence of IF

We identified 75 patients with *Fusarium* spp. isolated from clinical samples at 18 hospitals in Spain (Appendix Table 1) during 2000–2015. We excluded 10 patients with superficial infections and 7 with probable cases. In this study, the overall incidence of IF during 2000–2015 was 0.55 cases/100,000 admissions, corresponding to 0.42 neutropenic and 0.13 nonneutropenic patients/100,000 admissions (p<0.01). The overall incidence of IF was 0.40 cases/100,000 admissions during 2000–2009 and 0.79 cases/100,000 admissions during 2010–2015 (p<0.01). Among neutropenic patients, the incidence of IF increased from 0.32 to 0.57 cases/100,000 admissions (p = 0.06). Among nonneutropenic patients, the incidence of IF increased from 0.08 to 0.22 cases/100,000 admissions (p = 0.05). We also determined the annual cumulative incidence curves for the 2 groups of patients (Appendix Figure 1).

### Clinical Characteristics

We identified 58 cases of IF: 44 (75.9%) occurred in neutropenic patients and 14 (24.1%) in nonneutropenic patients (Table 1). Most (59%) patients were male. The median age was 67 years (interquartile range [IQR] 38–19 years) in neutropenic patients and 45 years (IQR 28–65 years) in nonneutropenic patients (p = 0.05). In total, 36.4% of neutropenic and 42.9% of nonneutropenic patients had received corticosteroid therapy in the previous 30 days (p = 0.66); 72.7% of neutropenic and 35.7% of nonneutropenic patients had received antifungal therapy in the previous 30 days (p = 0.66). At the end of follow-up (i.e., 90 days after diagnosis), 23 (52.3%) neutropenic patients had persistent neutropenia.

**Table 1:** Clinical characteristics of patients with invasive fusariosis, Spain, 2000–2015*

Variables	Total	Nonneutropenic	Neutropenic	p value†
Total	58 (100.0)	14 (100.0)	44 (100.0)	
Sex				
M	34 (58.6)	8 (57.1)	26 (59.1)	0.9
F	24 (41.4)	6 (42.9)	18 (40.9)	0.9
Median age, y (IQR)	51 (31–67)	67 (38–79)	45 (28–65)	0.05‡
Treatment history (previous 30 d)				
Antifungal	37 (63.8)	5 (35.7)	32 (72.7)	0.01
Corticosteroid	22 (37.9)	6 (42.9)	16 (36.4)	0.66
Persistent neutropenia	23 (39.7)	0	23 (52.3)	
Concurrent conditions				
Hematologic malignancy	46 (79.3)	5 (35.7)	41 (93.2)	<0.01§
Acute myeloid leukemia	20 (34.5)	1 (7.1)	19 (43.2)	
Acute lymphoid leukemia	8 (13.8)	0	8 (18.2)	
Non-Hodgkin lymphoma	5 (8.6)	2 (14.3)	3 (6.8)	
Aplastic anemia	4 (6.9)	0	4 (9.1)	
Multiple myeloma	3 (5.2)	2 (14.3)	1 (2.3)	
Myelodysplasia	2 (3.4)	0	2 (4.5)	
Chronic lymphoid leukemia	2 (3.4)	0	2 (4.5)	
Chronic myeloid leukemia	1 (1.7)	0	1 (2.3)	
Hodgkin´s lymphoma	1 (1.7)	0	1 (2.3)	
History of hematopoietic stem cell transplant	14 (24.1)	3 (21.4)	11 (25.0)	1.00§
Allogenic	9 (15.5)	1 (7.1)	8 (18.2)	
Autologous	4 (6.9)	2 (14.3)	2 (4.5)	
Cord blood haploidentical	1 (1.7)	0	1 (2.3)	
Graft-versus-host disease	5 (8.6)	1 (7.1)	4 (9.1)	1.00§
Acute	3 (5.2)	0	3 (6.8)	
Chronic	2 (3.4)	1 (7.1)	1 (2.3)	
Solid tumor	2 (3.4)	1 (7.1)	1 (2.3)	0.43§
History of solid organ transplant	4 (6.9)	3 (21.4)	1 (2.3)	0.04§
Other¶	10 (17.2)	5 (35.7)	1 (2.3)	<0.01§
Clinical manifestations				
Fever	48 (82.8)	8 (57.1)	40 (90.9)	<0.01§
Skin lesions	32 (55.2)	3 (21.4)	29 (65.9)	<0.01
Lung involvement	41 (70.7)	9 (64.3)	32 (72.7)	0.74§
Sinusitis	9 (15.5)	1 (7.1)	8 (18.2)	0.43§
Blindness	4 (6.9)	0	4 (9.1)	0.56§
Concurrent infection	31 (53.4)	6 (42.9)	25 (56.8)	0.36
Bacterial	17 (29.3)	2 (14.3)	15 (34.1)	0.18§
Fungal	4 (6.9)	1 (7.1)	4 (9.1)	0.56§
Viral	6 (10.3)	3 (21.4)	3 (6.8)	0.15§
Polymicrobial	4 (6.9)	1 (7.1)	3 (6.8)	1.00§
Type of fusariosis				
Localized	19 (32.8)	10 (71.4)	9 (20.5)	<0.01§
Cutaneous, localized	3 (5.2)	1 (7.1)	2 (4.5)	1.00§
Pneumonia	8 (13.8)	5 (35.7)	3 (6.8)	0.02§
Sinusitis	1 (1.7)	0	1 (2.3)	1.00§
Fungemia	7 (12.1)	4 (28.6)	3 (6.8)	0.05§
Disseminated	39 (67.2)	4 (28.6)	35 (79.5)	<0.01§
Diagnosis				
Culture	31 (53.4)	12 (85.7)	19 (43.2)	<0.01
Culture and histopathology	25 (43.1)	2 (14.3)	23 (52.3)	0.01
Histopathology	2 (3.4)	0	2 (4.5)	1.00

*Values are no. (%), except where indicated. †p values obtained by χ^2^ test, except where indicated.  ‡Determined by Mann-Whitney U test.  §Determined by Fisher exact test.  ¶Other conditions reported include hemophagocytic lymphohistiocytosis, chronic cardiac disease, T-cell prolymphocytic leukemia, rheumatoid arthritis, chronic obstructive pulmonary disease, and infantile respiratory distress syndrome.

In total, 41 (93.2%) neutropenic and 5 (35.7%) nonneutropenic patients had hematologic malignancies (p<0.01). In the neutropenic patient group, the most common hematologic malignancies were acute myeloid leukemia (43.2%) and acute lymphoid leukemia (18.2%). The proportion of patients who had received hematopoietic stem cell transplants (HSCTs) was 25% (11/44; 8 allogeneic, 2 autologous, and 1 cord blood haploidentical) in neutropenic patients versus 21.4% (3/14; 1 allogeneic and 2 autologous) in nonneutropenic patients (p = 1.00). Four (9.7%) neutropenic patients and 1 (7.1%) nonneutropenic patient had graft-versus-host disease (p = 1.00). Overall, 21.4% of nonneutropenic patients and 2.3% of neutropenic patients had a history of solid organ transplantation (p = 0.04). Finally, 35.7% of nonneutropenic and 2.3% of neutropenic patients had other underlying conditions (p = 0.04). Five nonneutropenic patients had chronic cardiac disease, T-cell prolymphocytic leukemia, rheumatoid arthritis, chronic obstructive pulmonary disease, or infantile respiratory distress syndrome; 1 neutropenic patient had hemophagocytic lymphohistiocytosis.

Neutropenic patients were more likely than nonneutropenic patients to have a fever at the time of diagnosis (90.9% vs. 57.1%; p<0.01). Neutropenic patients also were more likely to have skin lesions (65.9% vs. 21.4%; p<0.01). We did not observe a difference in the proportion of patients with lung involvement in the IF infection (72.7% of neutropenic vs. 64.3% of nonneutropenic patients; p = 0.74). We also did not observe a difference in the rate of concurrent infections (56.8% of neutropenic vs. 42.9% of nonneutropenic patients; p = 0.36) (Appendix Table 2). Disseminated disease occurred more frequently among neutropenic patients (79.5%) than nonneutropenic patients (28.6%; p<0.01). In contrast, localized forms of IF, especially localized pneumonia (6.8% of neutropenic patients vs. 35.7% of nonneutropenic patients; p = 0.02) and fungemia (6.8% of neutropenic patients vs. 28.6% of nonneutropenic patients; p = 0.05), were more common among nonneutropenic patients. However, disseminated fungemia was more common among neutropenic than nonneutropenic patients (45.7% vs. 25%; p = 0.03). Among neutropenic patients, skin lesions were more common in persons with disseminated IF (77.1%) than localized IF (22.2%; p = 0.004); lung involvement also was more common among those with disseminated IF (82.9% vs. 33.3%; p = 0.007).

Nonneutropenic patients were more likely than neutropenic patients to have a diagnosis on the basis of positive culture alone (43.2% of neutropenic patients vs. 85.7% of nonneutropenic patients; p<0.01). Neutropenic patients were more likely to have a diagnosis on the basis of a positive culture and histopathology (52.3%) than nonneutropenic patients (14.3%; p = 0.01).

### Isolated *Fusarium* Species

Thirty-six patients (62.1%) received a species-level etiologic diagnosis using molecular methods: 26 (44.8%) by genetic sequencing and 10 (17.2%) by MALDI-TOF mass spectrometry (Table 2). Among these patients, *F. solani* species complex (SC) was the most common (18/58; 31.0%), along with *Gibberella fujikuroi* SC (10/58; 17.2%), and *F. oxysporum* SC (5/58; 8.6%). The remaining 22 (37.9%) cases were identified as caused by *Fusarium* spp. Among disseminated infections, most (74.4%) were reported to species level; the most common were *F. solani* SC (15/39; 38.5%), *F. fukikuroi* SC (8/39; 20.5%), and *F. oxysporum* SC (3/39; 7.7%). In the cohort, disseminated IF also was caused by 2 *F. brachygibbosum* infections and 1 *F. dimerum* SC infection. Most (63.2%) localized infections were caused by *Fusarium* spp. *F. solani* SC (2/39; 5.1%), *F. fukikuroi* SC (2/39; 5.1%), and *F. oxysporum* SC (3/39; 7.7%) were the most common etiologic agents.

**Table 2:** Distribution of isolated *Fusarium* species, Spain, 2000–2015*

Species	Total	Localized	Disseminated	p value†
Total	58 (100.0)	19 (100.0)	39 (100.0)	
*F. solani* SC	18 (31.0)	3 (15.8)	15 (38.5)	0.08‡
*Gibberella fujikuroi* SC	10 (17.2)	2 (10.5)	8 (20.5)	0.29
*F. proliferatum*	6 (10.3)	0	6 (15.4)	0.08
*F. verticillioides*	3 (5.2)	2 (10.5)	1 (2.6)	0.25
*F. fujikuroi*	1 (1.7)	0	1 (2.6)	0.67
*F. oxysporum* SC	5 (8.6)	2 (10.5)	3 (7.7)	0.53
*F. brachygibbosum*	2 (3.4)	0	2 (5.1)	0.45
*F. dimerum* SC	1 (1.7)	0	1 (2.6)	0.67
*Fusarium* spp.	22 (37.9)	12 (63.2)	10 (25.6)	<0.01

*Values are no. (%), except where indicated. All identifications (except undefined *Fusarium* spp.) obtained by genetic sequencing or matrix-assisted laser desorption/ionisation time-of-flight mass spectometry. SC, species complex. †p values obtained by Fisher exact test, except where indicated.  ‡Determined by χ^2^ test.

### Therapeutic Regimens

Overall, 62.1% of patients received monotherapy (29.3% azoles, 32.8% amphotericin B), 27.6% of patients received combination therapy (e.g., azoles plus amphotericin B, mostly liposomal amphotericin B plus voriconazole), and 10.4% of patients did not receive an active treatment (3 patients were not treated, 2 received empirical caspofungin, and 1 received empirical micafungin) (Appendix Table 3). Nonneutropenic patients were more likely than neutropenic patients to receive voriconazole monotherapy (54.5% vs. 85.7%; p<0.01). We did not observe a difference in the proportions of patients who were treated with amphotericin B (36.4% of neutropenic patients vs. 21.4% of nonneutropenic patients; p = 0.35). Sixteen (36.4%) neutropenic patients received combination therapy: 14 received a lipid formulation of amphotericin B plus voriconazole and 2 received amphotericin B plus posoconazole. No nonneutropenic patients received combination therapy.

### Outcomes

By 90 days after diagnosis, 56.9% of all patients had died: 28.6% of nonneutropenic patients and 65.9% of patients with neutropenia at IF onset (p = 0.01). We analyzed these results using unadjusted Kaplan–Meier survival graphs (Appendix Figure 2; p = 0.03 by log-rank test). The death rate of nonneutropenic patients (28.6%) was similar to that of patients who had recovered from neutropenia (38.1%; p = 0.56) and both rates were significantly lower than among patients with persistent neutropenia (91.3%; p<0.01).

After 90 days, 66.7% of patients with localized and 36.8% with disseminated disease had died (p = 0.03). Overall, 66.7% (2/3) of patients with localized disease of the skin/soft tissue, 50% (4/8) with lung involvement, and 14.3% (1/7) with fungemia died.

In the assessment of prognostic factors, we analyzed 50 IF patients who received active antifungal therapy. In univariate analysis, 90-day death risk was associated with neutropenia at onset of IF and persistent neutropenia. Age, sex, age-adjusted Charlson comorbidity index, corticosteroid therapy in the previous 30 days, administration of antifungal in the previous 30 days, hematologic malignancy, history of HSCT, fungemia, disseminated disease, and the treatment type (i.e., monotherapy with a lipid formulation of amphotericin B, monotherapy with azoles or combined therapy) were not associated with 90-day survival. In multivariate analysis, persistent neutropenia (hazard ratio [HR] 7.08, 95% CI 1.91–26.17; p<0.01) was significantly associated with 90-day death risk (Table 3). We calculated adjusted Kaplan–Meier survival curves for the Cox regression model (Figure; area under the receiver operating characteristic curve = 0.83).

**Table 3:** Univariate and multivariate Cox regression analyses for 90-day death rate in 50 patients treated for invasive fusariosis, Spain, 2000–2015*

Characteristic	Unadjusted hazard ratio (95% CI)	p value	Adjusted hazard ratio (95% CI)	p value
Sex				
M	1.08 (0.52–2.25)	0.84	0.87 (0.41–1.84)	0.87
F	0.93 (0.45-1.93)	0.84		
Median age, y (IQR)	1.00 (0.99–1.01)	0.94		
Charlson index	1.01 (0.86–1.20)	0.87	1.16 (0.96–1.40)	0.12
Treatment history (previous 30 d)				
Antifungal	1.83 (0.81–4.14)	0.15		
Corticosteroid	0.69 (0.33–1.45)	0.33		
Neutropenia at onset	3.16 (0.95–10.44)	0.06		
Neutropenia at the end of follow-up				
Nonneutropenia	Referent		Referent	
Recovery from neutropenia	1.47 (0.38–5.69)	0.58	1.91 (0.48–7.64)	0.35
Persistent neutropenia	5.62 (1.65–19.11)	0.006	9.27 (2.43–35.42)	0.001
Hematologic malignancy	2.44 (0.74–8.06)	0.14		
History of hematopoietic stem cell transplant	1.39 (0.65–2.99)	0.4		
Fungemia	0.21 (0.03–1.54)	0.13		
Disseminated disease	1.90 (0.77–4.67)	0.16		
Antifungal therapy				
Monotherapy with lipid formulation of amphotericin B	1.95 (0.92–4.12)	0.08		
Monotherapy with azoles	0.47 (0.19–1.15)	0.10		
Combined therapy	1.08 (0.50–2.33)	0.84		

*Area under the receiver operating characteristic curve for this model was 0.82.

**Figure:**
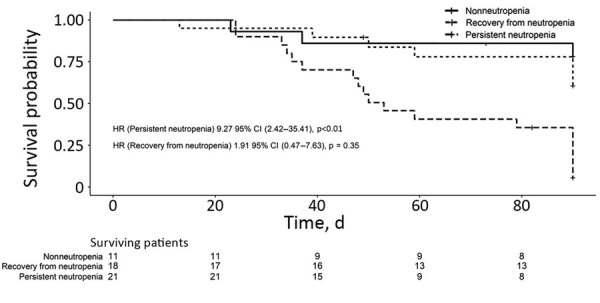
Adjusted Kaplan-Meier curves obtained from the stratified Cox regression model for 90-day survival in 50 patients treated for invasive fusariosis, Spain, 2000–2015. HR, hazard ratio.

## Discussion

We described 58 cases of invasive *Fusarium* infections at 18 hospitals in Spain during a 15-year period (2000–2015). During this time, IF incidence increased from 0.40 (2000–2009) to 0.79 (2010–2015) cases per 100.000 admissions (p<0.01). Incidence of IF in neutropenic patients increased from 0.32 to 0.57 cases per 100,000 admissions (p = 0.06). We observed a 3-fold increase in the incidence of IF in nonneutropenic patients, from 0.08 to 0.22 cases per 100,000 admissions (p = 0.05). This increase might have been caused by an increase in the at-risk population, environmental exposure to *Fusarium* conidia, the increased use of antifungal prophylaxis, or a combination of these factors.

To date, the highest IF incidence rates worldwide are in healthcare centers in Brazil. A cohort of HSCT recipients at hospitals in Brazil during 1985–2001 had a 0.6% prevalence of IF (*1*). During 2007–2009, ≈10 years later, a prospective multicenter study by Nucci et al. reported a 3.7% IF prevalence among allogeneic HSCT patients and 3.4% among patients with acute myeloid leukemia (*7*). That study also found a 1-year cumulative incidence of 5.2% among those who had received an allogeneic HSCT and 3.8% among those with acute myeloid leukemia or myelodysplasia (*7*). A >10-fold increase, from 86 to 1,023 IF cases per 100,000 admissions, occurred among patients with hematologic malignancies at a healthcare center in Brazil from 2000–2005 to 2006–2010 (*5*). In the United States, a prospective multicenter study conducted by the Transplant-Associated Infection Surveillance Network during 2001–2006 reported a 1-year cumulative incidence of up to 0.3% of non-*Aspergillus* mold infections (*23*). In Italy, a multicenter retrospective study reported a 0.2% incidence of IF among patients who had received an allogenic HSCT during 1999–2003 (*24*).

In this cohort in Spain, IF occurred frequently in patients with hematologic conditions and in HSCT recipients. IF occurs almost entirely in markedly immunosuppressed patients who usually are neutropenic; we identified IF in nonneutropenic patients, including patients with a history of solid organ transplants, chronic cardiac or lung disease, or rheumatoid arthritis. These findings are in agreement with Park et al. (*9*), who studied 37 patients with IF and noted that 54.1% of IF patients were nonneutropenic, 83.8% had hematologic malignancies, and 16.2% had history of solid organ transplantation (*9*). Thus, nonneutropenia and certain nonhematologic conditions might not be uncommon among IF patients.

Lungs, the bloodstream, and the skin were the organs most frequently affected by IF. *Fusarium* species produce aleuroconidia, yeastlike structures that can invade the bloodstream and might cause fungemia and metastatic skin lesions (*25*).

IF is associated with high death rates. This cohort had a 90-day death rate of 56.9%, similar to rates noted in other studies (*3*,*4*,*12*,*26*). The death rates of nonneutropenic and neutropenic patients who recovered from neutropenia were similar (28.6% of nonneutropenic patients vs. 38.1% of neutropenic patients; p = 0.56), consistent with previous reports that 70% of IF cases resolve when patients recover from neutropenia (*27*). In this cohort, patients with persistent neutropenia had a 91.3% death rate despite antifungal therapy. Persistent neutropenia was the single most predictive prognostic factor in IF, consistent with previous reports (*2*).

Researchers must identify effective therapeutic strategies to improve the prognosis of IF patients with persistent neutropenia. The treatment practices observed in our study align with guidelines from the European Society of Clinical Microbiology and Infectious Diseases Fungal Infection Study Group and European Confederation of Medical Mycology (*13*). We examined the effects of different therapeutic regimens on 90-day death rate. We found no differences in the outcome of patients treated with voriconazole and those receiving a lipid formulation of amphotericin B. Combination therapy also was not associated with outcome, consistent with results from the largest study series conducted so far (*3*,*12*). However, these results must be interpreted with caution. Nucci et al. (*3*) found that patients receiving combination therapy had more severe disease (*3*), possibly influencing the clinician’s decision to administer 2 drugs. In our study, no nonneutropenic patients received combination therapy; further studies should examine the potential benefits of combination therapy in nonneutropenic patients. Finally, because we observed similar clinical responses in patients treated with a lipid formulation of amphotericin B or voriconazole as monotherapies, clinicians might not need to rely on antifungal susceptibility tests to guide treatment. Most clinically relevant *Fusarium* isolates exhibit high minimal inhibitory concentrations to most antifungals, including azoles, echinocandins, and polyenes (*28*–*30*).

Our study is subject to the limitations of retrospective observational studies. For example, the sample size was limited to the cases with available data. Despite being a multicenter study during a 15-year period, the sample size might be insufficient to detect significant differences in some groups. Furthermore, clinicians might prescribe a more potent treatment regimen for patients with severe disease, possibly skewing our analysis of treatment outcomes. Although all IF cases in this retrospective study met standard criteria (*16*,*17*), we could not determine the causative species in every case. Thus, the *Fusarium* species was only reported when determined by genomic sequencing or MALDI-TOF mass spectrometry, because these techniques have high agreement rates (89.8%–97.0%) (*31*–*33*). All remaining IF cases in our series were reported as *Fusarium* spp. We also were not able to culture all *Fusarium* isolates because of a lack of specimens, especially for cases occurring before 2010 in hospitals in which *Fusarium* isolates were not collected as part of routine procedures

In conclusion, our data show that IF is an emerging infection in Spain. We report an increase in the incidence of IF among nonneutropenic patients, including those with hematologic conditions and other concurrent conditions, such as chronic cardiac or lung diseases, rheumatoid arthritis, or history of solid organ transplants. Our results support previous studies reporting that IF survival is critically dependent on resolution of neutropenia. 

Appendix Additional information on invasive fusariosis in nonneutropenic patients, Spain, 2000–2015.
