# Supplementary material for: Invasive Fusariosis in Nonneutropenic Patients, Spain, 2000–2015
Source: Emerg Infect Dis. 2021 Jan;27(1):26–35. doi: 10.3201/eid2701.190782 (PMC7774531; doi:10.3201/eid2701.190782)
Supplement: Appendix — Additional information on invasive fusariosis in nonneutropenic patients, Spain, 2000–2015. [file 19-0782-Techapp-s1.pdf]

Article DOI: https://doi.org/10.3201/eid2701.190782

Invasive Fusariosis in Nonneutropenic Patients, Spain, 2000–2015

Appendix

Appendix Table 1. Participating hospitals in study on invasive fusariosis, Spain, 2000–2015

Hospital
Hospital Universitario La Paz, Madrid
Hospital General Universitario Gregorio Marañón, Madrid
Hospital Universitari i Politècnic la Fe, Valencia
Hospital Universitario Cruces, Baracaldo, Vizcaya
Hospital Universitario Marqués de Valdecilla, Santander
Hospital Universitario Miguel Servet, Zaragoza
Hospital Universitario Virgen del Rocío, Seville
Hospital Universitario General de Alicante, Alicante
Hospital Universitario Reina Sofía de Córdoba, Córdoba
Fundación Instituto Valenciano de Oncología (FIVO), Valencia
Hospital Universiatario de Bellvitge, Barcelona
Hospital Universitario de Gran Canaria Doctor Negrín, Las Palmas de Gran Canaria
Hospital Universitario Ramón y Cajal, Madrid
Hospital Universitario Virgen de Valme, Seville
Hospital de la Santa Creu i Sant Pau, Barcelona
Hospital Universitario 12 de Octubre, Madrid
Hospital Universitari Vall d'Hebron, Barcelona
Hospital Universitario Puerta del Mar, Cádiz

Appendix Table 2. Characteristics of patients with localized and disseminated invasive fusariosis, Spain, 2000–2015*

Neutropenia	Neutropenic			Nonneutropenic		
Variables	Localized	Disseminated	p value†	Localized	Disseminated	p value†
Total	9 (100.0)	35 (100.0)		10 (100.0)	4 (100.0)	
Sex						
M	6 (66.7)	20 (57.1)	0.72	6 (60.0)	2 (50.0)	1.00
F	3 (33.3)	15 (42.9)	0.72	4 (40.0)	2 (50.0)	1.00
Median age, y (IQR)	45 (28–65)	47 (29–65)	0.81‡	58 (26–79)	67 (56–79)	0.57‡
Treatment history (previous 30 d)						
Antifungal	4 (44.4)	28 (80.0)	0.09	2 (20.0)	3 (75.0)	1.00
Corticosteroid	2 (22.2)	14 (40.0)	0.31	4 (40.0)	2 (50.0)	1.00
Underlying conditions						
Hematologic malignancy	8 (88.9)	33 (94.3)	0.51	3 (30.0)	2 (50.0)	0.58
Hematologic stem cell transplant	1 (11.1)	10 (28.6)	0.41	1 (10.0)	2 (50.0)	0.18
Graft-versus-host disease	1 (11.1)	3 (8.6)	0.56	0	1 (25.0)	0.29
Solid tumor	0	1 (2.9)	1.00	1 (10.0)	0	1.00
Other	1 (11.1)	1 (2.9)	0.37	6 (60.0)	2 (50.0)	1.00
Clinical manifestations						
Fever	8 (88.9)	32 (91.4)	1.00	4 (40.0)	4 (100)	0.09
Skin lesions	2 (22.2)	27 (77.1)	<0.01	1 (10.0)	2 (50.0)	0.18
Lung involvement	3 (33.3)	29 (82.9)	<0.01	5 (50.0)	4 (100)	0.22
Fungemia	3 (33.3)	18 (51.4)	0.46	4 (40.0)	1 (25.0)	1.00
Sinusitis	0	8 (22.9)	0.04	0	1 (25.0)	0.29
Blindness	0	4 (11.4)	0.57	0	0	
Concurrent infection	6 (66.7)	19 (54.3)	0.71	3 (30.0)	3 (75.0)	0.25

*Values are no. (%), except where indicated.
†p values obtained by Fisher exact test, except where indicated.
‡Determined by Mann-Whitney U test.

Appendix Table 3. Therapeutic regimens used to treat patients with invasive fusariosis, Spain, 2000–2015*

Treatment	Total	Neutropenic	Nonneutropenic	p value†
Total	58 (100.0)	44 (100.0)	14 (100.0)	
Monotherapy	36 (62.1)	24 (54.5)	12 (85.7)	0.04‡
Azole	17 (29.3)	8 (18.2)	9 (64.3)	0.002
Voriconazole	16 (27.6)	8 (18.2)	8 (57.1)	0.01‡
Posaconazole	1 (1.7)	0	1 (7.1)	0.24
Amphotericin B	19 (32.8)	16 (36.4)	3 (21.4)	0.35
Liposomal amphotericin B	17 (29.3)	14 (31.8)	3 (21.4)	0.52
Amphotericin B lipid complex	1 (1.7)	1 (2.3)	0	1.00
Amphotericina B deoxicholate	1 (1.7)	1 (2.3)	0	1.00
Combined therapy	16 (27.6)	16 (36.4)	0	0.006
Liposomal amphotericin B plus voriconazole	13 (22.4)	13 (29.5)	0	0.03
Liposomal amphotericin B plus posaconazole	2 (3.4)	2 (4.5)	0	1.00
Amphotericin B lipid complex plus voriconazole	1 (1.7)	1 (2.3)	0	1.00
Other§	3 (5.2)	2 (4.5)	1 (7.1)	1.00
Not treated	3 (5.2)	2 (4.5)	1 (7.1)	1.00

*Values are no. (%), except where indicated.
†p values obtained by Fisher exact test, except where indicated.
‡Determined by χ^2 test.
§2 patients received empirical caspofungin and 1 received empirical micafungin.

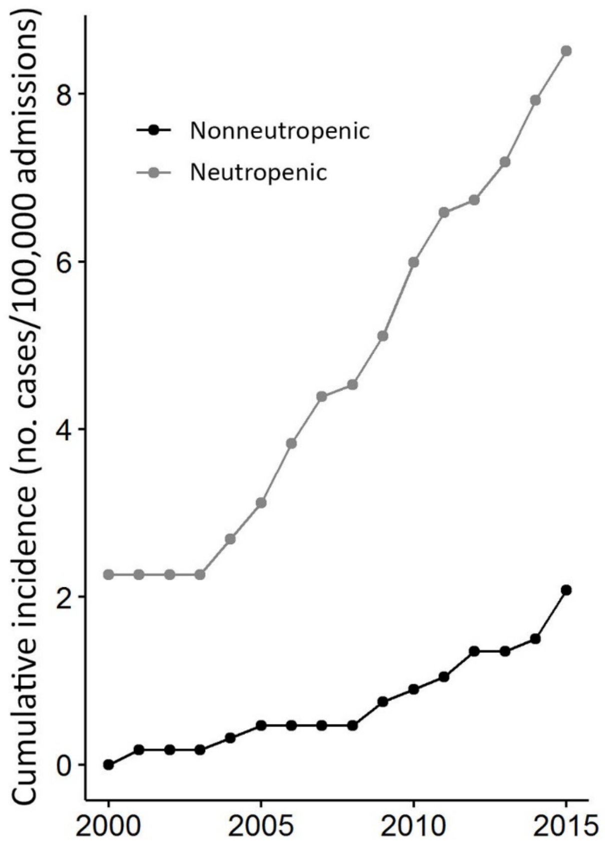

Appendix Figure 1. Yearly cumulative incidence of invasive infections caused by *Fusarium* spp., Spain, 2000–2015.

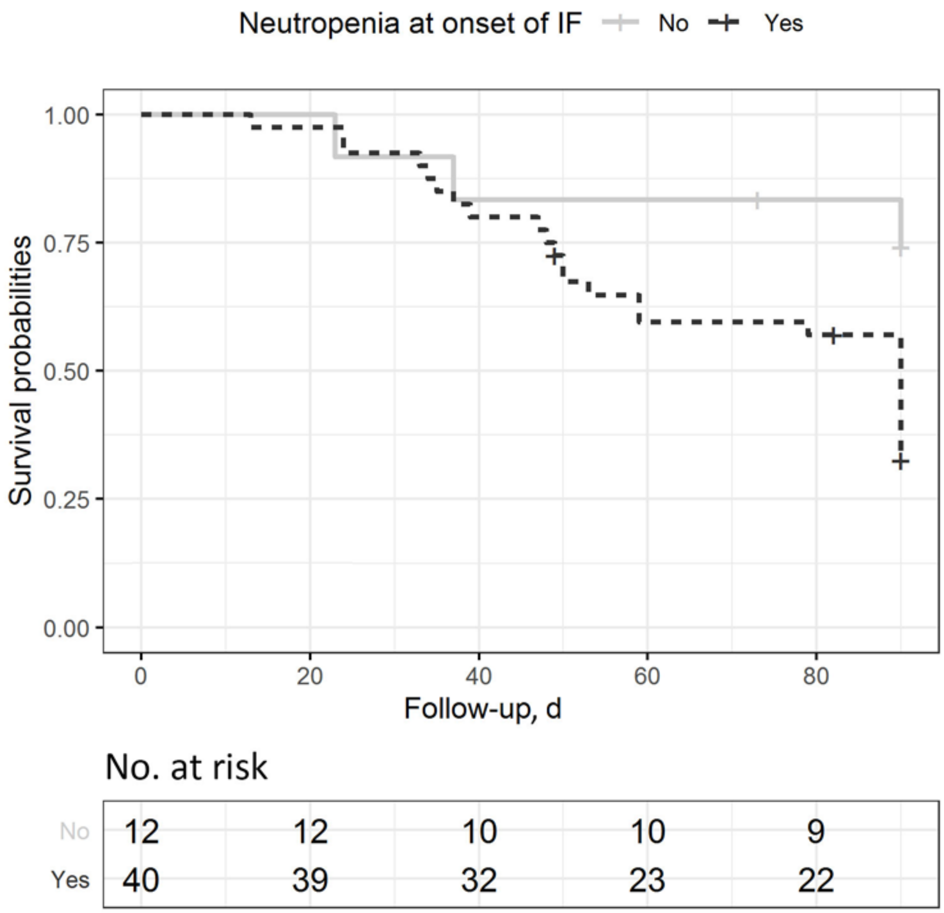

Appendix Figure 2. Probability of 90-day survival of patients with invasive fusariosis, Spain, 2000–2015. Log-rank test; $p = 0.03$.
